# Isolation and Characterization of Antibacterial Compounds from *Aspergillus fumigatus:* An Endophytic Fungus from a Mangrove Plant of the Sundarbans

**DOI:** 10.1155/2022/9600079

**Published:** 2022-04-22

**Authors:** S. M. Neamul Kabir Zihad, Md. Towhid Hasan, Mst Sabiha Sultana, Sushmita Nath, Lutfun Nahar, Mohammad A. Rashid, Shaikh Jamal Uddin, Satyajit D. Sarker, Jamil A. Shilpi

**Affiliations:** ^1^Pharmacy Discipline, Life Science School, Khulna University, Khulna 9208, Bangladesh; ^2^Department of Pharmacy, State University of Bangladesh, Dhaka 1205, Bangladesh; ^3^Guangzhou Institutes of Biomedicine and Health, Chinese Academy of Sciences, Guangzhou 510530, China; ^4^University of Chinese Academy of Sciences, Beijing 100049, China; ^5^Agrotechnology Discipline, Life Science School, Khulna University, Khulna 9208, Bangladesh; ^6^Centre for Natural Products Discovery (CNPD), School of Pharmacy and Biomolecular Sciences, Liverpool John Moores University, Byrom Street, Liverpool L3 3AF, UK; ^7^Laboratory of Growth Regulators, Institute of Experimental Botany ASCR, Palacký University, Šlechtitelů 27, Olomouc 78371, Czech Republic; ^8^Department of Pharmaceutical Chemistry, Faculty of Pharmacy, University of Dhaka, Dhaka 1000, Bangladesh

## Abstract

The Sundarbans, a UNESCO world heritage site, is one of the largest mangrove forests in one stretch. Mangrove plants from this forest are little studied for their endophytic fungi. In this study, we isolated fourteen endophytic fungi from the plants *Ceriops decandra* and *Avicennia officinalis* collected from the Sundarbans. Five of them were identified as *Aspergillus* sp. and one as *Penicillium* sp. by macroscopic and microscopic observation. Antibacterial activity of the crude extracts obtained from these endophytes was determined against *Staphylococcus aureus*, *Micrococcus luteus*, *Escherichia coli*, and *Pseudomonas aeruginosa* using resazurin-based microtiter assay. The isolated endophytes showed varying degrees of antibacterial activity with MICs ranging between 5 and 0.078 mg/mL. Molecular identification of the most active endophyte revealed its identity as *Aspergillus fumigatus* obtained from the leaves of *C*. *decandra*. Acute toxicity study of the ethyl acetate extract of *A*. *fumigatus* in mice revealed no mortality even at the highest dose of 2000 mg/kg bodyweight, though some opposing results are found in the subacute toxicity study. The extract was subjected to silica gel and Sephadex column chromatography resulting in the isolation of three pure compounds. LC-MS analysis of these pure compounds revealed their identity as fumigaclavine C, azaspirofuran B, and fraxetin. This is the first report of fraxetin from *A*. *fumigatus*. All three identified compounds were previously reported for their antibacterial activity against different strains of both Gram-positive and Gram-negative bacteria. Therefore, the observed antibacterial activity of the ethyl acetate (EtOAc) extract of *A*. *fumigatus* could be due to the presence of these compounds. These results support the notion of investigating fungal endophytes from the Sundarbans for new antimicrobial compounds.

## 1. Introduction

Despite the tremendous progress in therapeutics, there has always been a pressing need for the discovery of new and more effective antimicrobial agents in order to fight the continuously emerging multidrug-resistant pathogens. In addition to aging, immune deficiency, surgical interventions, and globalization have become the key contributors to the rapid spread of infections produced by multidrug-resistant microbes causing drastic increase in mortality, morbidity, and healthcare costs. Thus, search for new antimicrobial lead is a top priority of researchers and indeed microbes are the center of attention for their inherent ability to produce antibiotics. During the golden era of microbial product screening, researchers have introduced vast majority of microbial metabolites. However, many geographical locations still remain unexplored due to the difficulty in collecting samples from such locations. Many of the natural resources are studied partially and may render more bioactive compounds if further investigated. Therefore, the scientific community suggests the searching of known and new natural sources with broader diversity in antiinfective drug discovery programs [1–4].

For thousands of years, fungi have been a crucial part of human life. With current technological advances, utilizations of fungi have been extended to the production of enzymes, antibiotics, and pharmaceutical leads [5]. Both random and targeted screening has resulted in numerous fungal-derived antiinfective agents during the last few decades. But, nowadays, researchers are emphasizing on considering the ecological niche for new drug discovery, since the metabolic interaction between the fungi and its surroundings has a great influence on the production of secondary metabolites [6]. Fungi are found to reside within the intercellular spaces of plant roots, stems, leaves, and petioles that involve mutual metabolic interaction with no harm done to the host, rather rendering an indirect protection against herbivores [7–9]. Collectively, these fungi are known as endophytes, and they represent an important reservoir of different classes of bioactive compounds, especially antimicrobials [10]. The ground breaking discovery that introduced the endophytic fungi to researchers as a potential source of bioactive compound is the discovery of taxol, a billion-dollar drug from *Taxomyces andreanae* of Pacific yew tree (*Taxus brevifolia*). The later story is the identification of numerous potential lead compounds from endophytic fungi, mainly antibiotics produced to fight invading predators and pathogens [11–13]. Besides antibiotics, endophytic fungi also found to produce several other pharmacologically important compounds such as 22-triene-3b-ol with antimycotic activity, cajanol and podophyllotoxin with anticancer activity, kaempferol and ergoflavin with anti-inflammatory activity, lectin with antioxidant activity, heptelidic acid with insecticidal activity, sydoxanthone A and B with immunosuppressive activity, and radicicol with cytotoxic activity [14–21].

At the beginning of the exploration of endophytic fungi for bioactive leads, researchers mainly focused on terrestrial plants. The role of surrounding environment on the production of unnatural secondary metabolites has prompted the researcher to endophytic fungi associated with plants and other living organisms of marine, mangrove, and regions with extreme environments. The Sundarbans of Bangladesh, a UNESCO world heritage site, is the largest coastal wetland forest in the world. Although the plants of the Sundarbans have undergone both pharmacological and phytochemical investigation to some extent, very little attention has been paid towards its endophytic diversity [22]. Due to the presence of a rich source of nutrients and the complex nutrient dynamics, mangroves are referred to as the homeland of microbes [23]. For this reason, several studies were conducted on the endophyte communities associated with mangrove plants found on the coastlines along the Pacific, Indian, and Atlantic oceans [24]. Another interesting fact is that the endophytes associated with mangrove plants represent a pool of marine, freshwater, and soil microbes as mangrove forests confer an interface between upland terrestrial and coastal estuarine ecosystems, thus presenting a great reservoir of bioactive natural products of wide diversity [25]. In Bangladesh, drug discovery from endophytic fungi associated with mangrove plants is new, and parts of the Sundarbans are still unexplored. The aim of our study was to explore the endophytic fungal community associated with two medicinal plants of the Sundarbans, namely, Goran (*Ceriops decandra*) and Baen (*Avicennia officinalis*), evaluate antibacterial activity of these endophytes, and to isolate and characterize the secondary metabolite produced by bioactive fungal endophyte.

## 2. Materials and Methods

### 2.1. Collection of Samples

Plant samples were collected from the Kolagachia range of the Sundarbans. Three types of plant parts (stem, bark, and leaf) belonging to two plant species, namely, Goran (*Ceriops decandra*) and Baen (*Avicennia officinalis*), were collected. Collection area was registered by the global positioning system (GPS) for the ease of documentation, publication, or recollection if necessary. The identity of the collected plants was confirmed by the experts of Bangladesh National Herbarium where voucher specimen was submitted for future reference (Table 1).

### 2.2. Isolation of Endophytic Fungi

At first, the plant parts were washed with sterile distilled water to remove unwanted debris. The plant parts were surface sterilized using 75% ethanol for 1 min, 0.5% sodium hydrochloride for 3 min, and 75% ethanol for 3 × 15 s, followed by thorough wash with sterile distilled water. Then, they were cut into small pieces about the size of 0.5 cm^2^ or 2 mm × 2.5 mm with a sharp sterile blade and placed horizontally on Petri dishes containing Sabouraud dextrose agar (SDA) medium. Streptomycin, at a concentration of 200 *µ*g/ml, was added to the SDA medium to inhibit bacterial growth. The Petri dishes were incubated at room temperature for 1–3 weeks to obtain fungal colony. Colonies with the same appearance, morphology, and color were considered as the similar species. To obtain a pure culture, a tiny pinch from a distinct colony was taken from 1st culture plate and inoculated into Petri dishes containing SDA medium amended with streptomycin. After obtaining a pure culture, the fungus was grown for 7–10 days [12, 26].

### 2.3. Morphological Identification

To observe the morphology of the isolated endophytic fungi, the cultures were grown on SDA plates for 7–10 days at 25°C. To begin the morphological identification process, glass slides and cover slips were cleaned using 95% ethanol. Afterwards, a drop of lactophenol cotton blue was placed onto a clean microscope slide. Then, a tiny pinch of the fungus taken from the culture plate was placed onto the dye, spread using the needle, and covered with a cover slip. Then, it was examined under the microscope, and the pictures were taken.

### 2.4. Extraction of Secondary Metabolites

For extraction of the secondary metabolites produced by the isolated endophytic fungi, they were first grown in broth media. To do so, the fungus from the pure culture plates were transferred to SDA slants and incubated for 48–72 h at 30°C to obtain the growth phase of the individual fungus. Then, the growing part of fungus from the SDA slant was transferred to Scott bottle containing potato dextrose broth media using a transfer loop. It was incubated in a cubic shaker (150 rpm) (ThermoStable Precise Shaking Incubator, Daihan Scientific, South Korea) at 28 ± 2°C for 21 days. Afterwards, crude extracts were obtained from the broth medium according to the method described by Li et al. [27]. At first, the mycelium was separated aseptically from broth by filtering through cotton plug and transferred to a separating funnel. The broth was defatted by partitioning with *n*-hexane followed by extraction with dichloromethane and ethyl acetate. Dichloromethane and ethyl acetate extracts were dried over anhydrous sodium sulfate and then evaporated by the rotary vacuum evaporator to get the extracts. The separated mycelium was dried, crushed using a mortar-pestle, and macerated in methanol for three days. The solvent was then separated from the marc and evaporated using the rotary evaporator to get methanol extract of the mycelium.

### 2.5. Antibacterial Screening

Resazurin-assisted microtiter plate-based antibacterial assay was used to investigate the antibacterial activity of the endophytic extracts, and the results were expressed in MIC [28]. Two Gram-positive (*Staphylococcus aureus* NCTC 12981 and *Micrococcus luteus* NCTC 7508) and two Gram-negative (*Escherichia coli* NCTC 12241 and *Pseudomonas aeruginosa* NCTC 12903) bacteria were used for the antibacterial screening. The bacteria were grown in nutrient broth at 37°C for 12–18 h and centrifuged afterwards at 4000 rpm for 5 min to get bacterial pellet which was suspended in 5 mL of sterile normal saline. Then, the McFarland standard was used to determine the bacterial concentration and adjusted to working concentration of 5 × 10^6^ cfu/mL by necessary dilution.

The extracts were prepared in 5% (v/v) DMSO at a concentration of 10 mg/mL. Then, they were serially diluted to get the concentration range of 0.078–10.00 mg/mL and added to the wells of a 96-well plate at a volume of 50 *μ*L. Then, 10 *μ*L of resazurin indicator solution was added to each well. Afterwards, 30 *μ*L of nutrient broth was added to each well. Finally, 10 *μ*L of bacterial suspension (5 × 10^6^ cfu/mL) was added to each well to achieve a concentration of 5 × 10^5^ cfu/mL. Then, the plates were incubated at 37°C for 18–24 h, and the color change was assessed visually after incubation. Change of color from purple to pink or colorless was recorded as positive, and the lowest concentration at which color change occurred was taken as the MIC value. Streptomycin with a concentration range of 0.005–0.640 mg/mL was used as the positive control in this study.

### 2.6. Molecular Identification of Antibacterial Endophytic Fungi

Molecular identification of the most active endophytic fungi was done according to the protocol described by Qadri et al. [12]. The fungal mycelia were freeze-dried, and the cells were lysed in 10 ml of extraction buffer. Afterwards, the lysate was extracted by adding an equal volume of isopentanol/chloroform (1 : 24), followed by centrifugation at 10000 × *g* for 10 min at 4°C, and the genomic DNA was precipitated from the aqueous phase in a 2 × volume of chilled isopropanol by centrifugation at 10000 × *g* for 10 min at 4°C. Then, the ITS4 and ITS5 regions of the genomic DNA were PCR amplified using universal ITS primers, ITS4 (5ʹTCCTCCGCTTATTGATATGC3′) and ITS5 (5ʹGGAAGTAAAAGTCGTAACAA3′). In PCR amplification, 50 *μ*L reaction volume contained 3 *μ*L (2 ng/*μ*L) of DNA, 10 *μ*L 5 × reaction buffer, 5 *μ*L of 10 mM dNTP, 3 *μ*L of 1.5 M MgCl_2_, 3 *μ*L of 100 pmol primers, and 26 *μ*L of distilled water. The PCR cycle was as follows: initial denaturation at 94°C for 5 min, 39 cycles at 94°C for 30 s for each denaturation, annealing of the primers at 51°C for 30 s, extension at 72°C for 2 min, and final extension at 72°C for 5 min and held at 4°C. Then, the amplified product was verified by 1.5% agarose gel electrophoresis followed by gel purification, sequencing, and matching with the sequences in the GenBank (NBLAST).

### 2.7. Acute and Subacute Toxicity Test

To study the acute toxicity of the extract of the most active endophytic fungus, young Swiss albino mice (age 2-3 weeks) with average weight of 31–36 g were purchased from Jahangirnagar University, Bangladesh, and kept in standard environmental condition for one week in the animal house of Pharmacy Discipline, Khulna University. Mice groups, each containing five mice, were orally administered with 2000, 1000, and 500 mg/kg bodyweight of aqueous extract following the method adopted by Makrane and colleagues [29]. The control group received vehicle (water) alone. The animals were observed continuously for first 24 h and 14 days for any signs of changes, mortality, and body weight.

The subacute toxicity test was carried out as per the procedure described by Johari et al. (2017) with some modifications [30]. The experimental animals were divided into the test group and control group, and each group comprised of five mice. The test group was administered with the test extract at a dose of 500 mg/kg b.w./p.o. for 14 days, while the control group received the vehicle only. After treatments, all the experimental animals were observed daily for any abnormal clinical signs and mortality for 14 days. At the end of the observation period, the animals were anaesthetized, and their blood samples were collected through cardiac puncture for biochemical studies. The blood samples were allowed to clot and centrifuged at 3000 rpm for 10 min, and serum was separated. Then, the serum levels of ALT (alanine transaminase), AST (aspartate transaminase), ALP (alkaline phosphatase), and TB (total bilirubin) were measured using a bioanalyzer (HumaLyzer Primus, HUMAN Gesellschaft für Biochemica und Diagnostica, Germany).

### 2.8. Chemical Composition Study

#### 2.8.1. Chromatographic Separation

Active extract was subjected to silica gel (60–120 mesh) open column chromatography. The sample was adsorbed onto small amount of silica gel and added to the top of the column. Gradient elution was carried out with different mixtures of *n*-hexane and chloroform (10 : 0 to 0 : 10). Fractions of about 10 mL each were collected in test tubes, and the solvent was evaporated for detection by TLC. Each fraction was spotted on the TLC plate with the developing solvent system consisting of different ratios of *n*-hexane and chloroform. Developed TLC plates were visualized under short (254 nm) and long (365 nm) wavelength of UV light and finally sprayed with vanillin/sulfuric acid reagent [31]. Fractions obtained from silica gel chromatography were pooled together on the basis of their TLC pattern and subjected to size exclusion chromatography using Sephadex LH20 (GE Healthcare Bioscience, Sweden). Prior to column packing, Sephadex LH20 was soaked in chloroform for overnight, and the elution was done with chloroform.

#### 2.8.2. Identification of Active Compounds

Identification of compounds was achieved by using LC-MS analysis performed on a 6530 Accurate-Mass *Q* TOF LC/MS system (Agilent Technologies, UK). A ZORBAX Eclipse Plus C18 Rapid Resolution HD (2.1 × 50 mm, 1.8 *μ*m) column was used to determine the molecular mass. The conditions were set as follows: column temperature at 25°C, UV-Vis detector at 200–400 nm, 0.4 mL/min of flow rate, and 20 *μ*L of injection volume with sample concentration of 10 ppm. An elution gradient was used with 0.1% formic acid in water as mobile phase A and 0.1% formic acid in methanol as mobile phase B. The mobile phase composition started from 5% B at 0 min and increased as linear gradient to reach 100% B at 13 min. An electrospray ion source was used with ionization mode set to positive. The response was recorded in real time by the mass spectrometer data system. The parameters were set as follows: electrospray interface 3000 V, sample cone 60 V, extraction cone 4 V, rangefinder lens 300 V, desolvation temperature 250°C, source temperature 100°C, nebulizer gas flow 20 L/h, desolvation gas flow 760 L/h, and TOF tube 4687 V. The data acquisition method was set as follows: cycle time 1 s, scan duration 0.9 s, interscan delay 0.1 s, mass range *m/z* 100–1700, and centroid mode. Results were processed using the Agilent MassHunter Qualitative Analysis software (version B.08.00), and compounds were identified by comparison with published respective mass spectral data found in mass spectral databases and published literature.

## 3. Results and Discussion

In this study, a total of 14 endophytic fungi were isolated from the selected plants. Among these, seven were isolated from *C*. *decandra* and seven were from *A*. *officinalis*. For convenience, these fungi were given codes and used subsequently in the manuscript (Table 2).

Isolated pure cultures of fungal isolates from the plant samples were further cultured on Petri dishes and left to grow for 7–10 days to get maturation stage. At this stage, the outer structure, colors of the mycelium and spores, as well as the pattern of mycelium formation were observed for morphological identification and macroscopic evaluation. Images of 7–10 days old pure cultures are given in the Supplementary Material (Figures S1–S5).

**Figure 1:**
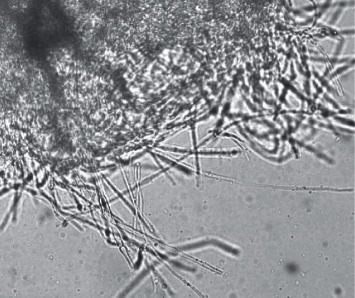
Microscopic image of GOL-1.

Morphological identification of fungi usually involves both macroscopic and microscopic observations. Macroscopic evaluation involves observing the appearance of the colony, the texture of mycelia, and pigmentation on both sides of the culture plate, whereas microscopic evaluation involves observing the conidia, conidiophore, and branching pattern [32]. Endophytic fungi isolated from different parts of *C*. *decandra* and *A*. *officinalis* showed distinct colony appearance and pigmentation. The microscopic images of the isolated fungi are shown in Figure 1 and Supplementary Material (Table S1). GOL-2, GOB-1, GOS-3, BAS-3, and BAS-4 were identified as *Aspergillus* sp. as they showed fast growing, white to yellow-brown to black colonies, and conidiophores terminating in a vesicle covered with differently arranged phialides [33]. Whereas, GOS-1 was identified as *Penicillium* sp. presenting fast growing colonies in shades of white and green. It consisted of a dense felt of conidiophores with multiple branching [33]. The rest of the isolated endophytic fungi remain unidentified through microscopic evaluation.

The resazurin-based antibacterial assay used in this study is a modified protocol that utilizes a standard concentration of bacterial suspension which helps to obtain true MIC values [28]. This blue dye, resazurin, is reduced to a pink colored compound, resorufin by oxidoreductases present in viable cells, and it has been long utilized to demonstrate bacterial and yeast contamination of milk [34, 35]. The MIC values obtained for the isolated endophytic fungi against the selected Gram-positive and Gram-negative bacteria are given in Table 3. The MIC values ranged between 0.078 and 10 mg/mL against four strains of bacteria used in this study. Streptomycin, used as the standard in this assay, was active against all four pathogens tested with MIC values ranging between 0.010 and 0.039 mg/mL.

Among the 16 isolates, GOL-1 (EtOAc), GOS-2 (DCM), and BAS-3 (EtOAc) showed promising wide spectrum of antibacterial activity. Among all the fungal isolates, extracts of GOL-1 and GOS-2 were the most active with MIC values of 78 *µ*g/mL against some of the pathogens tested.

The PCR amplified and sequenced ITS region of the genomic DNA of the fungal isolate was matched using nucleotide database BLASTN. Prior to database search, necessary amendments were made to the original sequence to conform to the database annotation conventions that included trimming off strings of N's, low quality sequence, vector or linker from the end, correction of feature spans and modification of descriptions for coding regions, and adjustment of exon spans to conform to the splice donor/acceptor consensus sequences, GT and AG, respectively. Matching with the existing records, GOL-1 was identified as *Aspergillus fumigatus* due to 98.99% similarity in identity (Table 4). The sequence data were submitted to GenBank, and an accession number was obtained (OL989212).

Due to its strong antibacterial activity as well as identification through genome matching, GOL-1 was further selected for the bioactivity study. In the acute toxicity test, no mortality was observed after the administration of the fungal extract GOL-1 even at the highest dose of 2000 mg/kg bodyweight. Apart from mortality, we also observed several other parameters to evaluate whether the extract produced any immediate toxic effect in the test animals including food intake, skin color, drowsiness, sedation, eye color, diarrhea, and coma. No abnormality was found in all the three dose groups (500, 1000, and 2000 mg/kg b.w.) of mice. In the subacute toxicity assay, serum levels of three organ specific marker enzymes, namely, ALT, AST, and ALP were measured in the test animals after 14-day administration of the fungal extract. Serum levels of ALT and AST are two established biomarkers of hepatic condition as they are leaked into blood after any damage occurs to the liver. In addition to hepatic damage, serum AST serves as a nonspecific damage marker of heart muscle. ALP level is another marker enzyme that represents liver dysfunction [36]. Total bilirubin also serves as a biomarker for liver function and integrity. A rise in total bilirubin (TB) level resembles hepatic dysfunction [37]. In our study, we found some contradictory results while assessing these parameters. The levels of serum ALT and AST were elevated in mice administered with the test extract compared to the control animals indicating liver dysfunction/damage (Table 5). Whereas, serum ALP and TB values in the test group were found very close to the values found in the control mice, thus indicating that the test extract is not likely to cause much hepatic damage (Table 5). Thus, these results remained inconclusive to ascertain any hepatotoxic role of the ethyl acetate extract of GOL-1 in test animals.

**Figure 2:**
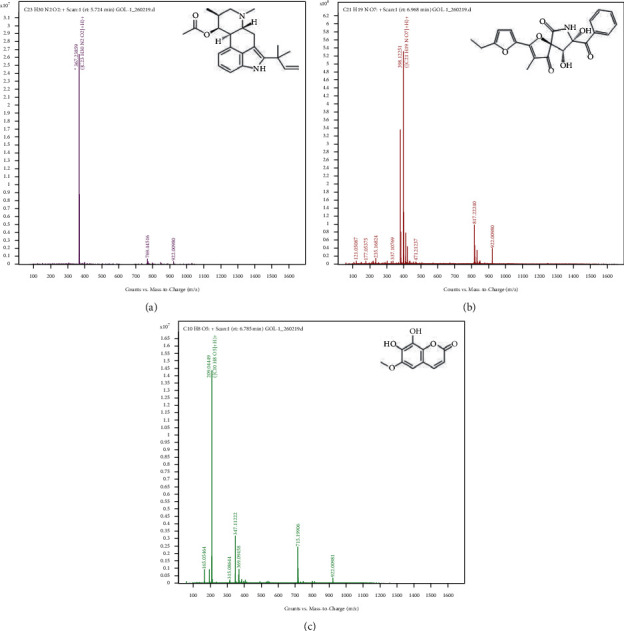
Mass spectra and structure of the identified compounds: (a) fumigaclavine C, (b) azaspirofuran B, and (c) fraxetin.

The ethyl acetate extract of GOL-1 was subjected to silica gel open column chromatography for separation of secondary metabolites, and the fractions thus obtained were subjected to TLC to detect the presence of compounds. Based on the TLC pattern, eluted fractions were pooled together to give three final fractions which were subjected to Sephadex column chromatography for further purification. Purification of these three fractions resulted in the isolation of three pure compounds which were identified through LC-MS analysis. Based on the LC-MS data and available literature, these three compounds were identified as fumigaclavine C, azaspirofuran B, and fraxetin (Table 6 and Figure 2).

The Sundarbans comprises a diverse ecosystem with the existence of dynamic relations between its members to maintain the integrity of this system. The biodiversity of this forest is composed of numerous species of microorganisms, planktons, invertebrates, amphibians, mollusks, and mammals. Due to the abundance of organic matter and nutrients, microorganisms constitute a major portion of the biomass of Sundarbans. Of the total microbial community, bacteria and fungi constitute 91%, while the rest is constituted by algae and protozoa [38]. Despite this plenteousness, very few works have been done on the manglicolous fungi of this forest. Some pioneering works have been done in recent years which reported the antibacterial potential of endophytic fungi associated with the plants of Sundarbans. One such study led to the identification of kojic acid, oxysporone, and xylitol with profound antibacterial potential [39–42]. In this study, we isolated and identified three compounds produced by endophytic *A*. *fumigatus* associated with *C*. *decandra*, which showed profound activity in the antibacterial assay. Among these three compounds, fumigaclavine C and azaspirofuran B were previously isolated from *A*. *fumigatus*. Fumigaclavine C was identified in *A*. *fumigatus* as an endophyte of *Cynodon dactylon* stem, while azaspirofuran B was identified in *A*. *fumigatus* collected from the Red Sea sediment in Hurghada, Egypt [43, 44]. Fumigaclavine C, isolated from marine derived *A*. *fumigatus*, showed cytotoxic activity against MCF-7 breast cancer cells with the underlying mechanism of apoptosis [45]. This alkaloid has also been reported to have antibacterial activity against *S*. *aureus*, *P*. *aeruginosa*, *E*. *coli*, and *Bacillus subtilis* [46], anticolitis [47], anti-inflammatory [48], aortic ring relaxant [49], and hepatoprotective activities [50]. In addition to *A*. *fumigatus*, azaspirofuran B was also isolated from the fermentation culture of another *Aspergillus* species and *Aspergillus sydowii* [51, 52]. This heterocyclic *γ*-lactam derivative has been reported to have antitumor [51], cytotoxic [52], and antiseizure activities [44]. El-Hady et al. reported tyrosinase inhibitory, acetylcholinesterase inhibitory, antioxidant, and antimicrobial activities of sponge derived *A*. *sydowii* and suggested these activities could be due to azaspirofuran B, which is a major compound of that extract [53]. This is the first report of fraxetin from *A*. *fumigatus*. This compound has been reported previously from the plant *Lawsonia inermis* [54] and showed antibacterial activity against *S*. *aureus* [55] and antifungal activity against *Trichophyton tonsurans*, *T*. *rubrum*, and *T*. *mentagrophytes* [56]. Fraxetin showed its antibacterial activity by increasing cell membrane permeability. It also caused the disruption of nucleic acid and protein synthesis by inhibiting the binding of topoisomerase with DNA [55]. This compound has diverse pharmacological activities including anticancer [57], antitumor, antimetastatic [58], neuroprotective [59], hepatoprotective [60], antioxidant [61], antihyperglycemic [62], and antifibrotic activities [63].

## 4. Conclusion

This study revealed three bioactive compounds, namely, fumigaclavine C, azaspirofuran B, and fraxetin, in the broth culture extract of the endophytic fungus *A*. *fumigatus* isolated from the leaves of *C*. *decandra*. Fumigaclavine C and azaspirofuran B have been reported previously from *A*. *fumigatus* for their diverse pharmacological potentials. This is the first report of fraxetin from endophytic *A*. *fumigatus*. Previous reports of antibacterial activity of these three compounds provide the support for the observed antibacterial activity of the ethyl acetate extract of *A*. *fumigatus*. In conclusion, the present investigation dictates the importance of bioassay-guided investigation of fungal endophytes from the Sundarbans for antimicrobial drug discovery. It clearly reveals that the fungal endophytes are rich in natural product content and requires intensive attention for the discovery of new chemical scaffolds with antibacterial potential.

## Figures and Tables

**Table 1:** Name and geographic location of the plant samples.

Local name	Scientific name	Family	Voucher specimen number	Collected part (s)^*∗*^	Place of collection^†^
Goran	*Ceriops decandra*	Rhizophoraceae	(DACB30322)	L, B, S	KG (22.2152°N, 89.2376°E)
Baen	*Avicennia officinalis*	Acanthaceae	(DACB35541)	S, B	KG (22.2151°N, 89.2368°E)

L = leaf; B = bark; S = stem; ^†^KG, Kolagachia forest range, Munshiganj, Satkhira.

**Table 2:** Isolated endophytic fungi from the collected mangrove plants.

Plant	Part	Number of isolates	Code name
*Ceriops decandra*	Leaves	2	GOL-1, GOL-2
	Bark	2	GOB-1, GOB-2
	Stem	3	GOS-1, GOS-2, GOS-3
*Avicennia officinalis*	Bark	2	BAB-1, BAB-2
	Stem	5	BAS-1, BAS-2, BAS-3, BAS-4, BAS-5

**Table 3:** Results of antibacterial screening of the isolated endophytes.

Endophytic fungi	Extract^*∗*^	MIC (mg/mL)^*∗∗*^			
		PA	EC	SA	MC
GOL-1	DCM	5.0	—	—	—
	EtOAc	0.156	0.156	0.625	0.078
	Et-OH (M)	2.5	—	2.5	5.0
GOL-2	DCM	—	—	—	—
	EtOAc	1.25	1.25	0.625	0.625
	Et-OH (M)	—	—	—	—
GOB-1	DCM	0.156	5.0	—	—
	EtOAc	1.25	5.0	1.25	1.25
	Et-OH (M)	—	—	—	—
GOB-2	DCM	—	—	5.0	—
	EtOAc	0.625	1.25	0.625	0.625
	Et-OH (M)	—	—	—	—
GOS-1	DCM	—	—	1.25	—
	EtOAc	0.625	0.625	1.25	0.625
	Et-OH (M)	—	—	—	—
GOS-2	DCM	0.078	0.078	0.156	0.156
	EtOAc	—	—	—	—
	Et-OH (M)	—	—	—	—
GOS-3	DCM	—	—	—	—
	EtOAc	—	—	—	—
	Et-OH (M)	—	—	—	—
BAB-1	DCM	—	—	—	—
	EtOAc	—	—	—	—
	Et-OH (M)	—	—	—	—
BAB-2	DCM	5.0	—	—	—
	EtOAc	0.625	0.312	0.625	0.625
	Et-OH (M)	—	—	—	—
BAS-1	DCM	5.0	—	—	—
	EtOAc	10	—	—	—
BAS-2	DCM	—	—	—	—
	EtOAc	0.625	0.625	0.156	0.325
	Et-OH (M)	—	—	1.25	—
BAS-3	DCM	0.312	—	—	—
	EtOAc	—	—	—	—
	Et-OH (M)	—	—	—	—
BAS-4	DCM	1.25	—	—	—
	EtOAc	2.5	1.25	5.0	—
	Et-OH (M)	—	—	—	—
BAS-5	DCM	1.25	2.5	0.156	0.625
	EtOAc	0.625	1.25	1.25	1.25
	Et-OH (M)	—	—	—	—
Streptomycin	—	0.010	0.019	0.039	0.019

^*∗*^DCM, dichloromethane; EtOAc, ethyl acetate; Et-OH (M), ethanol extract of myceli. ^*∗∗*^PA, *P*. *aeruginosa* (NCTC 12903); EC, *E*. *coli* (NCTC 12241); SA, *S*. *aureus* (NCTC 12981); MC, *M*. *luteus* (NCTC 7508).

**Table 4:** Result of molecular identification of GOL-1.

DNA sequence	Identification	Query cover	% Identity	Reference (blastn)	GenBank accession number
CTTTGGAAGTAAAAAATGTAACAAGGTTTCCGTAGGTGAACCTGCGGAAGGATCATTACCGAGTGAGGGCCCTCTGGGTCCAACCTCCCACCCGTGTCTATCGTACCTTGTTGCTTCGGCGGGCCCGCCGTTTCGACGGCCGCCGGGGAGGCCTTGCGCCCCCGGGCCCGCGCCCGCCGAAGACCCCAACATGAACGCTGTTCTGAAAGTATGCAGTCTGAGTTGATTATCGTAATCAGTTAAAACTTTCAACAACGGATCTCTTGGTTCCGGCATCGATGAAGAACGCAGCGAAATGCGATAAGTAATGTGAATTGCAGAATTCAGTGAATCATCGAGTCTTTGAACGCACATTGCGCCCCCTGGTATTCCGGGGGGCATGCCTGTCCGAGCGTCATTGCTGCCCTCAAGCACGGCTTGTGTGTTGGGCCCCCGTCCCCCTCTCCCGGGGGACGGGCCCGAAAGGCAGCGGCGGCACCGCGTCCGGTCCTCGAGCGTATGGGGCTTTGTCACCTGCTCTGTAGGCCCGGCCGGCGCCAGCCGACACCCAACTTTATTTTCTAAGGTGACCTCGATCAGTAGGTGCCACTTTCCC	*Aspergillus fumigatus*	99%	98.99%	MG674663.1	OL989212

**Table 5:** Values of serum ALT, AST, ALP, and TB levels in test animals.

Sample (dose)	ALT (U/L)	AST (U/L)	ALP (U/L)	TB (mg/dl)
GOL-1 extract (500 mg/kg b.w.)	158.92 ± 13.01	230.50 ± 18.50	205.34 ± 14.13	0.38 ± 0.10
Control	46.67 ± 6.58	40.17 ± 1.17	204.00 ± 8.83	0.43 ± 0.03

**Table 6:** LC-MS results of compounds isolated from *A*. *fumigatus* extract.

Sample	Type of sample	Molecular formula for quasimolecular ion	Observed *m/z*	Representative compound
*A*. *fumigatus*	EtOAc extract	([C_23_H_30_N_2_O_2_]+H)^+^	367.23859	Fumigaclavine C
		([C_21_H_19_NO_7_]+H)^+^	398.12251	Azaspirofuran B
		([C_10_H_8_O_5_]+H)^+^	209.04449	Fraxetin

## Data Availability

The datasets used in this study are available from the corresponding author upon request.

## Supplementary Materials

Supplementary Materials Morphological and microscopic identification of isolated endophytic fungi (Table S1 and Figures S1–S5).
