# Supplementary material for: Isolation and Characterization of Antibacterial Compounds from Aspergillus fumigatus: An Endophytic Fungus from a Mangrove Plant of the Sundarbans
Source: Evid Based Complement Alternat Med. 2022 Apr 22;2022:9600079. doi: 10.1155/2022/9600079 (PMC9054444; doi:10.1155/2022/9600079)
Supplement: Supplementary Materials — Morphological and microscopic identification of isolated endophytic fungi (Table S1 and Figures S1–S5). [file 9600079.f1.docx]

# Supplementary material

**Table S1: Microscopic images and morphological identification of isolated endophytic fungi.**

| **Code of fungi** | **Microscopic image** | **Identification status** |
| --- | --- | --- |
| GOL-1 | 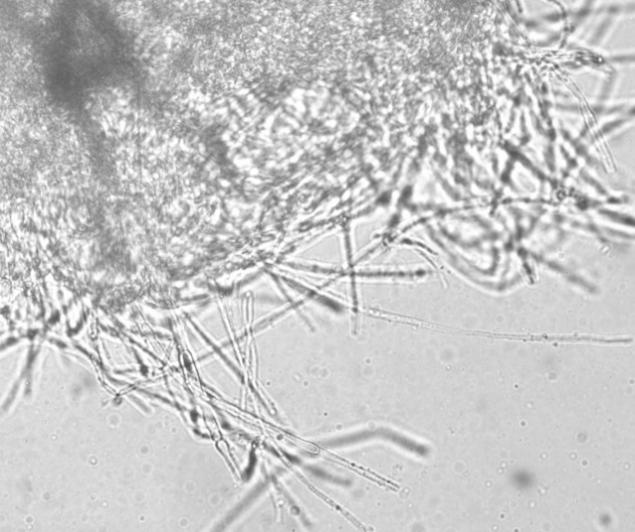 | *Aspergillus fumigatus* (identified through gene sequencing, see **Table 4**) |
| GOL-2 | 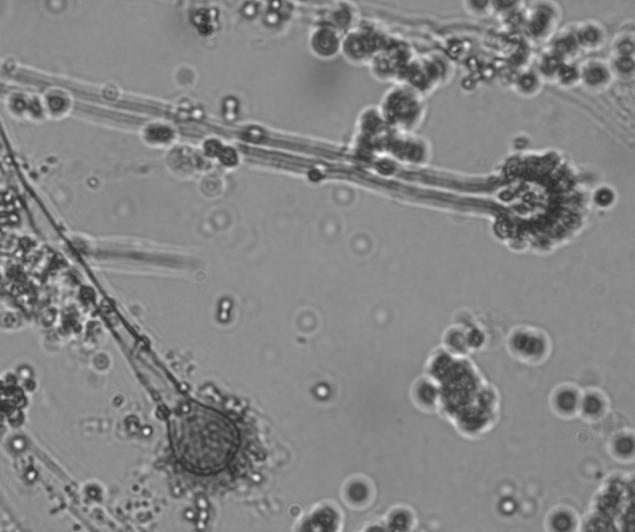 | *Aspergillus sp.*  Conidia |
| GOB-1 | 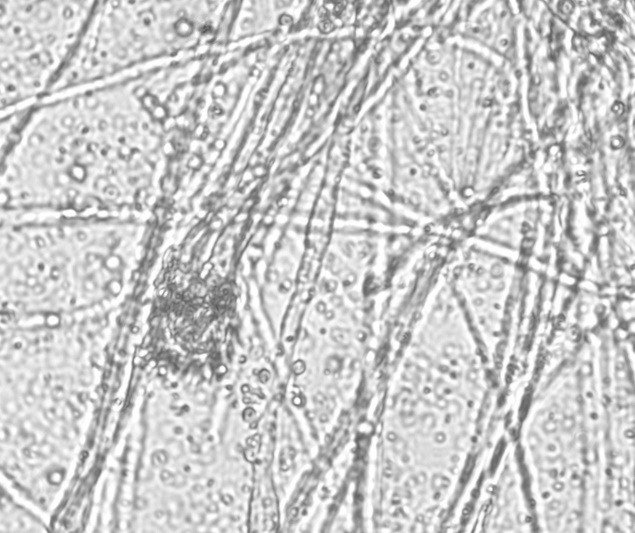 | *Aspergillus sp.*  Conidia |
| GOB-2 | 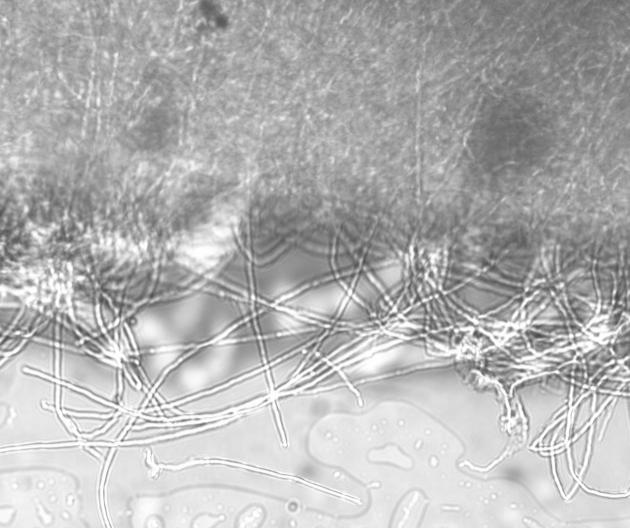 | Unidentified |
| GOS-1 | 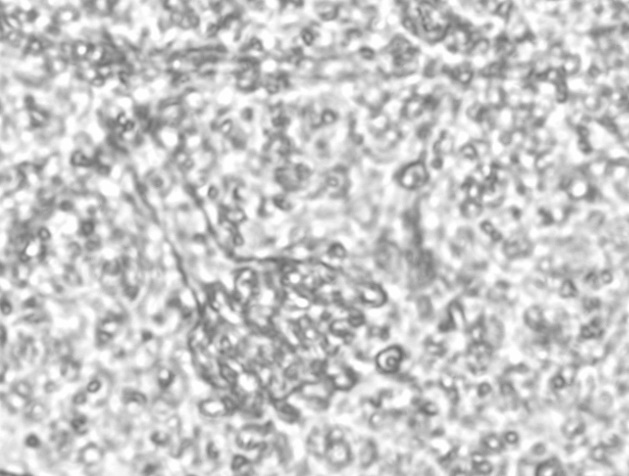 | *Penicillium sp.*  Conidia |
| GOS-2 | 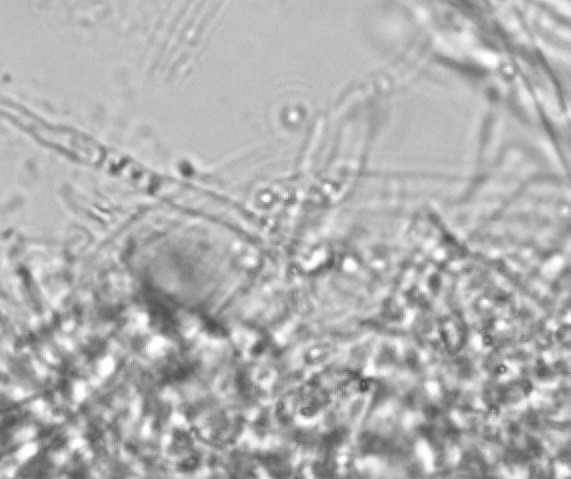 | Unidentified |
| GOS-3 | 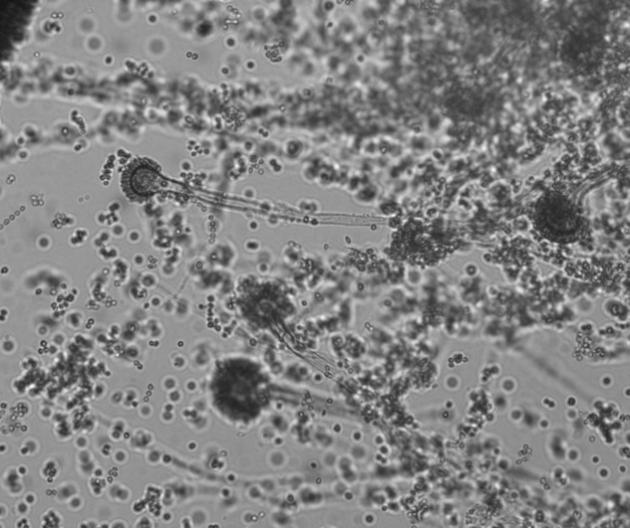 | *Aspergillus sp.*  Conidia |
| BAB-1 | 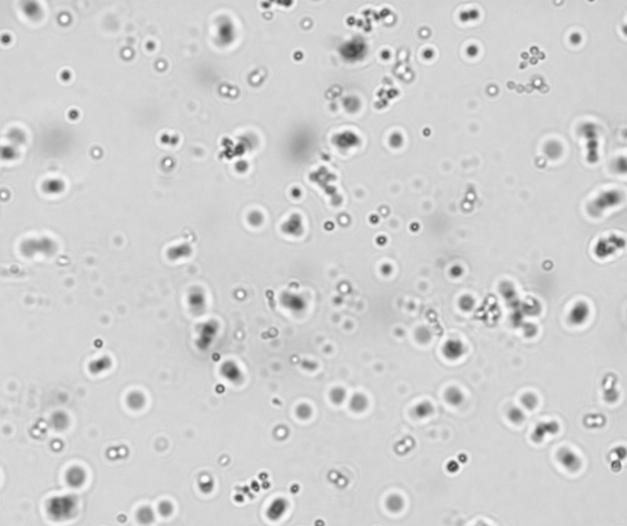 | Unidentified |
| BAB-2 | 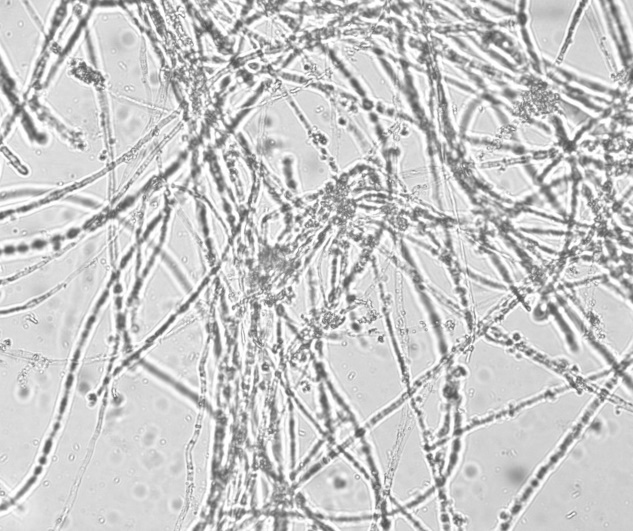 | Unidentified |
| BAS-1 | 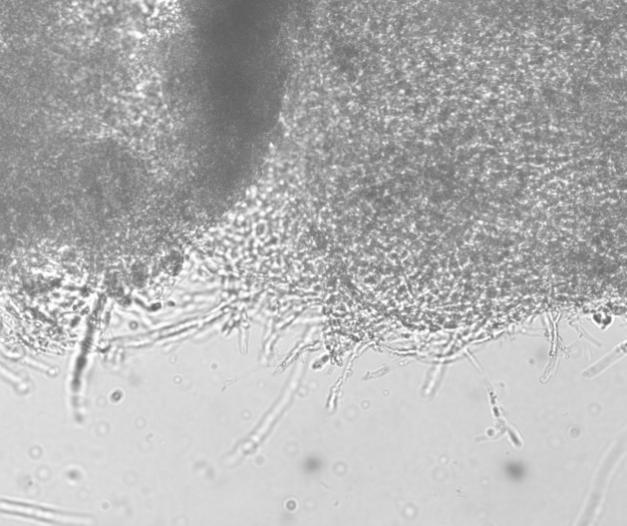 | Unidentified |
| BAS-2 | 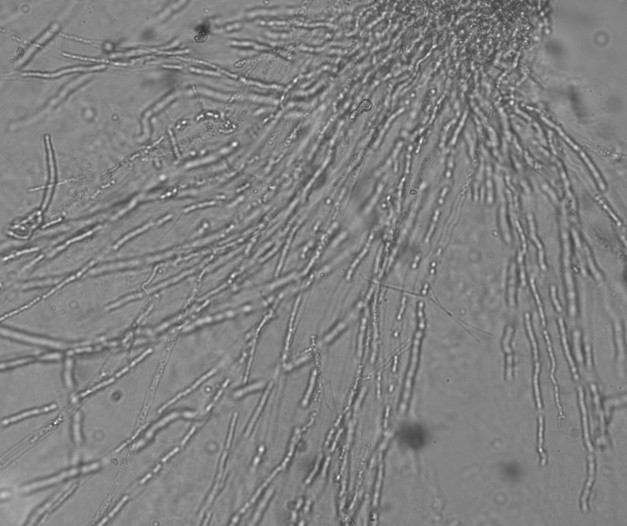 | Unidentified |
| BAS-3 | 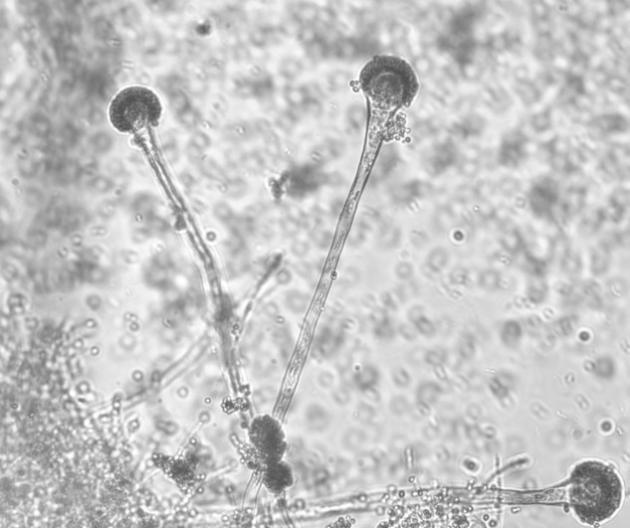 | *Aspergillus sp.*  Conidia |
| BAS-4 | 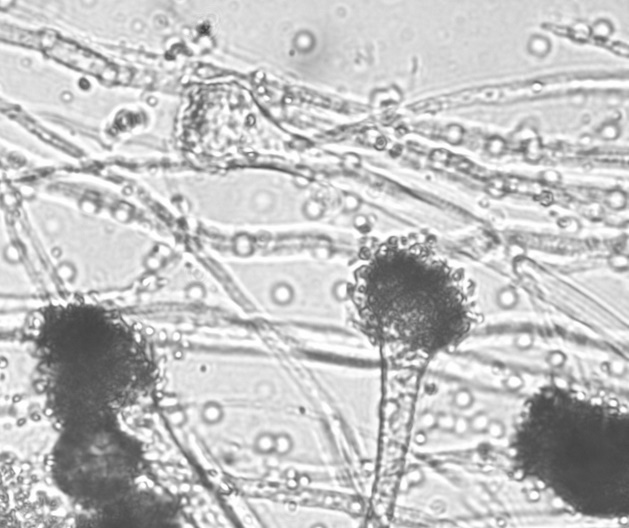 | *Aspergillus sp.*  Conidia |
| BAS-5 | 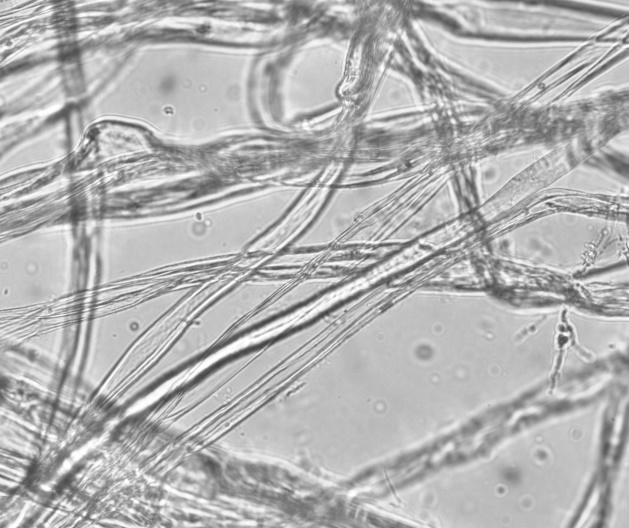 | Unidentified |


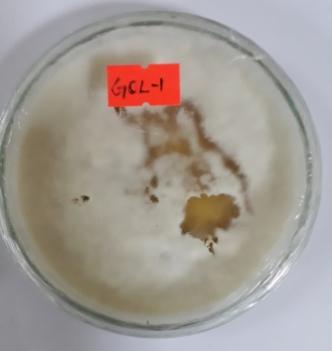

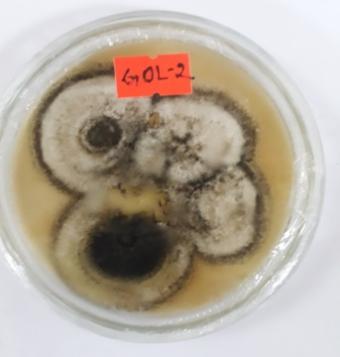


**Figure S1:** **Endophytes isolated from leaves of *Ceriops decandra.***


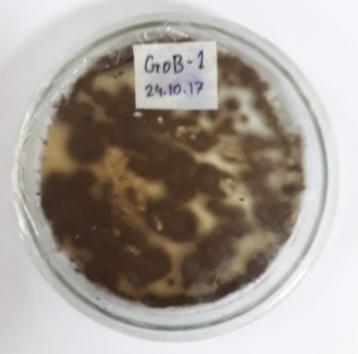
 ***
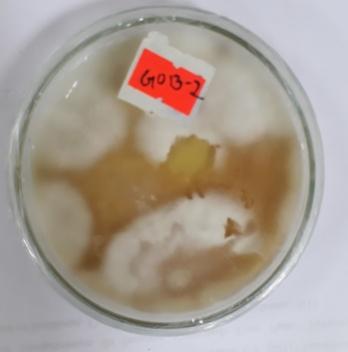
***

**Figure S2:** **Endophytes isolated from bark of *Ceriops decandra.***


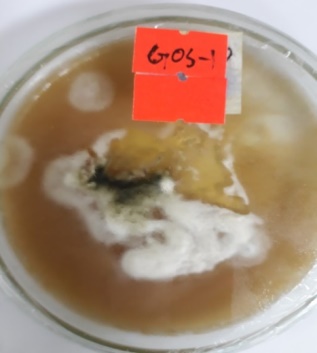
 ***
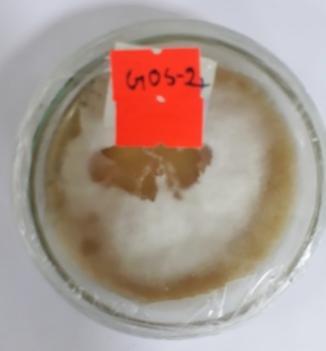
***
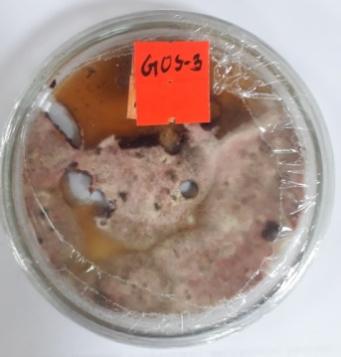


**Figure S3:** **Endophytes isolated from stem of *Ceriops decandra.***

***
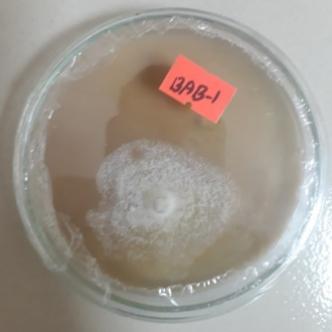

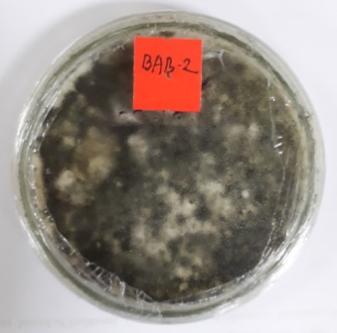
***

**Figure S4:** **Endophytes isolated from bark of *Avicennia officinalis.***

***
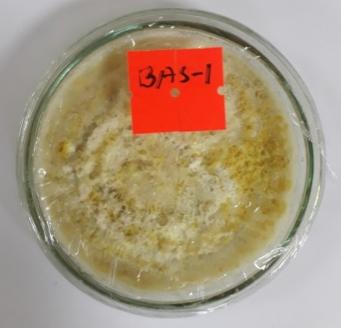

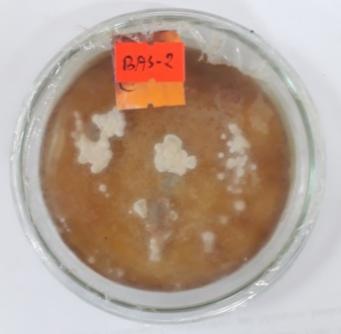

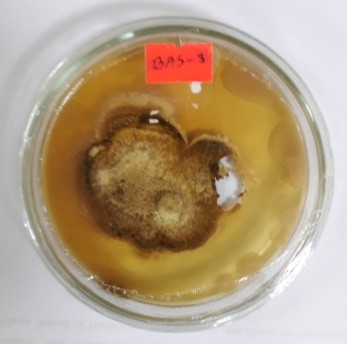
***

***
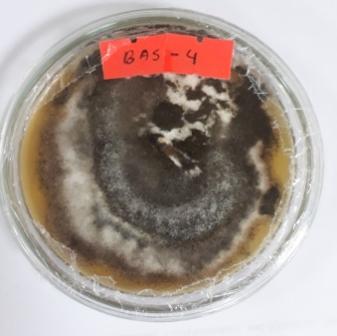

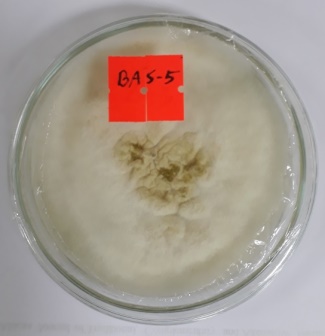
***

**Figure S5:** **Endophytes isolated from stem of *Avicennia officinalis.***
